# Translational regulation of *Anopheles gambiae* mRNAs in the midgut during *Plasmodium falciparum* infection

**DOI:** 10.1186/1471-2164-13-366

**Published:** 2012-08-02

**Authors:** Edward A Mead, Meng Li, Zhijian Tu, Jinsong Zhu

**Affiliations:** 1 Department of Biochemistry, Virginia Tech, 311 Engel Hall, Blacksburg, VA, 24061, USA; 2 Current address: Department of Animal Sciences, Rutgers University, New Brunswick, NJ, 08901, USA

**Keywords:** Genome-wide study, mRNA translation, Polysome, Mosquito immune reaction, Malaria parasite

## Abstract

**Background:**

Malaria is caused by *Plasmodium* parasites, which are transmitted via the bites of infected Anopheline mosquitoes. Midgut invasion is a major bottleneck for *Plasmodium* development inside the mosquito vectors. Malaria parasites in the midgut are surrounded by a hostile environment rich in digestive enzymes, while a rapidly responding immune system recognizes *Plasmodium* ookinetes and recruits killing factors from the midgut and surrounding tissues, dramatically reducing the population of invading ookinetes before they can successfully traverse the midgut epithelium. Understanding molecular details of the parasite-vector interactions requires precise measurement of nascent protein synthesis in the mosquito during *Plasmodium* infection. Current expression profiling primarily monitors alterations in steady-state levels of mRNA, but does not address the equally critical issue of whether the proteins encoded by the mRNAs are actually synthesized.

**Results:**

In this study, we used sucrose density gradient centrifugation to isolate actively translating *Anopheles gambiae* mRNAs based upon their association with polyribosomes (polysomes). The proportion of individual gene transcripts associated with polysomes, which is determined by RNA deep sequencing, reflects mRNA translational status. This approach led to identification of 1017 mosquito transcripts that were primarily regulated at the translational level after ingestion of *Plasmodium falciparum*-infected blood. Caspar, a negative regulator of the NF-kappaB transcription factor Rel2, appears to be substantially activated at the translational levels during *Plasmodium* infection. In addition, transcripts of Dcr1, Dcr2 and Drosha, which are involved in small RNA biosynthesis, exhibited enhanced associations with polysomes after *P. falciparum* challenge. This observation suggests that mosquito microRNAs may play an important role in reactions against *Plasmodium* invasion.

**Conclusions:**

We analyzed both total cellular mRNAs and mRNAs that are associated with polysomes to simultaneously monitor transcriptomes and nascent protein synthesis in the mosquito. This approach provides more accurate information regarding the rate of protein synthesis, and identifies some mosquito factors that might have gone unrecognized because expression of these proteins is regulated mainly at the translational level rather than at the transcriptional level after mosquitoes ingest a *Plasmodium*-infected blood meal.

## Background

Malaria is the most deadly tropical parasitic disease faced by mankind, responsible for around 1.2 million deaths each year [1]. The malaria parasite *Plasmodium* must complete a complex developmental cycle in the mosquito in order to be transmitted from person to person. When a female mosquito feeds on an infected human, it takes up parasite-laden blood. *Plasmodium* gametocytes rapidly differentiate to male and female gametes, and fertilize inside the mosquito midgut to produce zygotes. The zygotes develop into motile ookinetes that invade and traverse the midgut epithelial cells [2]. In the space between the midgut epithelium and the basal lamina, the ookinetes transform into oocysts. After maturation, each oocyst ruptures and sends out thousands of sporozoites into the hemolymph. These sporozoites later migrate to the mosquito’s salivary glands and are released into the saliva during a subsequent blood meal, infecting another person and completing the parasite cycle in the mosquito [3].

Mosquitoes have developed various mechanisms to confront *Plasmodium* infection. To accomplish transmission from person to person, the malaria parasite *Plasmodium* must undergo complex developmental transitions and survive numerous attacks from the mosquito’s innate immunity system [2,4]. A variety of mosquito factors have been shown to affect the development of *Plasmodium* parasites in the mosquito {reviewed in [4,5]}. A better understanding of the cellular and molecular mechanisms that underlie vector-parasite interaction may provide critical targets and facilitate the development of new effective malaria control strategies.

The midgut represents one of the most challenging environments for the survival and development of *Plasmodium*. Microarray analyses have unravelled many transcriptional changes in the mosquito midgut in response to *Plasmodium* infection [6-8]. These informative studies detail the relative abundance of steady-state mRNA, providing the vector-borne disease community with valuable information regarding the transcriptional response to infection. However, these assays could not address whether or when the cognate proteins are actually synthesized, and whether translational regulation of mosquito mRNA takes place in response to *Plasmodium* challenge.

Polysome profiling has been previously used in other organisms to identify translational regulation in nutritional homeostasis [9], cellular stress response [9,10], embryogenesis [11], spermatogenesis [12], cancer progression and cancer chemotherapy [13,14]. This approach is based on the principle that messenger RNAs that are being actively translated usually have multiple ribosomes associated with them, forming large structures known as polysomes. In contrast, translationally inactive mRNAs generally are associated with messenger ribonucleoprotein particles or a single ribosome (collectively called “nonpolysomes”). Sucrose density gradient centrifugation is used to separate polysomes from nonpolysomes. The mRNA levels of individual genes in polysome fractions and nonpolysome fractions are determined by using a variety of different methods, and the ratios reflect mRNA translational status [15]. Here we report our systematic study of the translational status of individual mRNA species in *Anopheles gambiae* midguts after a blood meal containing *Plasmodium falciparum* gametocytes. RNA polysomal profiling indicates that transcripts of a large group of mosquito genes are enriched in polysome fractions in *P. falciparum*-infected mosquitoes, as compared with the uninfected control. The result suggests that translational regulation of gene expression in mosquito midguts has a profound impact on the anti-malaria reactions. This approach provides more molecular details in the interaction between mosquitoes and malaria parasites, and may reveal additional targets that can be exploited to reduce mosquito vector competence via genetic manipulation.

## Results

### Changes in mRNA association with polysomes after *P. falciparum* infection

Our hypothesis is that midgut invasion by *P. falciparum* ookinetes alters mosquito gene expression at both the transcriptional and translational levels. To test this hypothesis, we analyzed genome-wide mRNA translational status by measuring the proportion of individual mRNA species in polysome complexes.

**Figure 1:**
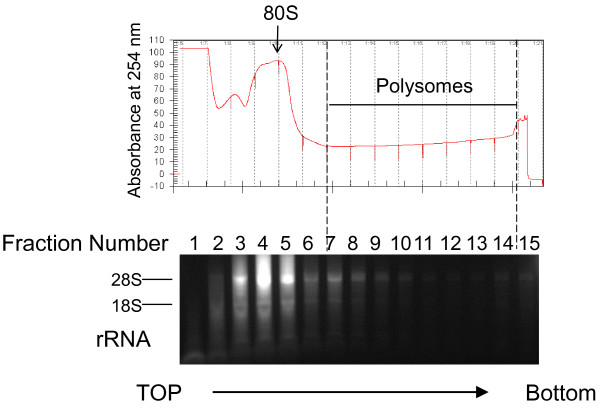
**Isolation of mRNA associated with polysomes.** Midguts were collected from female adult *An. gambiae* mosquitoes at about 24 h after ingestion of *P. falciparum*-infected or uninfected blood. Cellular extracts were sedimented by centrifugation in a 10-60% sucrose gradient, and 15 fractions were collected. Absorbance profile at 254 nm (top) is shown for a representative sample together with analysis of rRNA extracted from each fraction. RNA was separated on 1% agarose/formaldehyde gel followed by SYBR gold staining. The position of the 80 S peak is indicated.

Midguts from female *Anopheles gambiae* mosquitoes were dissected at approximately a day (22–26 hours) after ingestion of *P. falciparum*-infected blood. Mosquitoes fed on uninfected blood were used as control. A portion of the midguts were used for isolation of total cellular RNA. Extracts of the remaining midguts were fractionated over sucrose density gradients, and fifteen fractions were collected from the top of each gradient (Figure 1). Non-polysomal fractions, as well as polysomal fractions were combined, respectively, to obtain two RNA pools per gradient for three independent experiments. The first, last and the sample between non-polysomal and polysomal, were all discarded to ensure pure pools from each set. A fourth experiment was conducted where the polysomal and non-polysomal samples were not pooled, to be used later for qRT-PCR. The mRNA levels of individual mosquito genes in polysome (PS) fractions, nonpolysome (NP) fractions and unfractionated steady-state (total) RNA were determined using high throughput RNA deep sequencing. Each RNA pool generated 2.1-9.8 million raw reads (Table 1).

**Table 1:** ** *An. gambiae* RNA-seq Expression Data**

	**Infected Non-polysomal**	**Infected Polysomal**	**Uninfected Non-polysomal**	**Uninfected Polysomal**	**Uninfected Total RNA**	**Infected Total RNA**
Total Reads Set 1	7785361	4244042	4781123	4766035	2081115	6150051
Total Reads Set 2	3871849	3442387	3351084	2203276	9837495	4328298
Total Reads Set 3	6597022	6980908	8588835	5980648	3009585	8407264
Reads Mapped to Transcriptome Set 1	3527074 (45.3%)	3423900 (80.7%)	3608010 (75.5%)	1049208 (22.0%)	1483831 (71.3%)	5144550 (83.7%)
Reads Mapped to Transcriptome Set 2	2332634 (60.2%)	1476806 (42.9%)	2557909 (76.3%)	473146 (21.5%)	7772228 (79.0%)	2612416 (60.4%)
Reads Mapped to Transcriptome Set 3	4942339 (74.9%)	5191081 (74.4%)	7221237 (84.1%)	4992710 (83.5%)	2634845 (71.3%)	5992586 (71.3%)

Sequence reads are listed for three independent experiments (Sets 1–3). The six samples were multiplexed and sequenced together. Each set had roughly 30 million raw reads. Erroneous reads and reads where linkers were incomplete or missing were excluded from total number of reads. Reads mapped to transcriptome are the number (and percentage) of reads which matched back to the *An. gambiae* cDNA P3.4 database.

Signal intensities of transcripts from each PS pool were compared to those of transcripts from a matching NP pool. To quantify the translational status of individual mRNA species, we define the relative PS loading (PL) as the extent of mRNA association with polysomes:

(1) $$PL=\frac{Expression\;level\;in\;PS}{Expression\;level\;in\;NP+Expression\;level\;in\;PS}$$

PL values were compared between the mosquitoes fed on *P. falciparum*-infected or -uninfected blood. After exposure to *Plasmodium* parasites, 1170 transcripts became increasingly associated with polysomes (≥ 2 fold, *p* < 0.05) in mosquito midguts (Additional file 1: Table S1). In contrast, only 7 transcripts shifted more towards nonpolysomal fractions (≥ 2 fold, *p* < 0.05).

**Figure 2:**
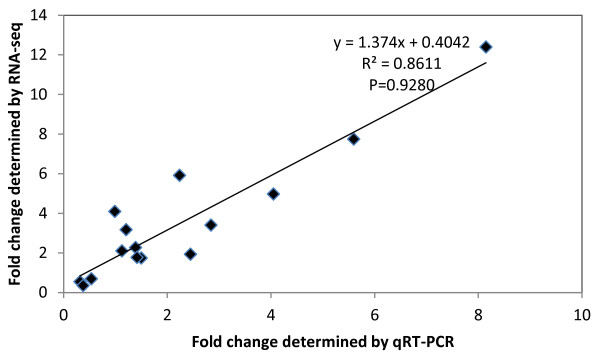
**Validation of the RNA-seq results with quantitative RT-PCR.** The extent of mRNA association with polysomes was compared between mosquitoes fed on *P. falciparum*-infected and uninfected blood. Changes in the relative PS loading (PL) obtained by RNA-seq analysis were plotted against the corresponding changes obtained with real-time RT-PCR. For both RNA-seq and RT-PCR analysis, the mean values of PL were calculated based on data from three independent experiments. Sixteen genes were analyzed. The Pearson correlation coefficient (p =0.9280) and the best-fit linear-regression analysis (R^2^ = 0.8611) demonstrated a high degree of correlation of the magnitude of regulation between the two assays.

To validate the alteration of polysome-mRNA association, we carried out quantitative real-time RT-PCR (qRT-PCR) analysis with PS and NP RNAs collected in the above-mentioned experiments. PL values were compared for 16 selected genes between the *Plasmodium*-challenged and control mosquitoes. These genes were selected to represent various expression levels and functional categories. A decent correlation coefficient (R^2^ = 0.8611) was observed between the PL values determined by RNA deep sequencing and qRT-PCR, indicating that RNA-seq analysis provides reliable PL data (Figure 2).

### Identification of mosquito genes that are primarily regulated at the translational level in response to *P. falciparum* infection

**Figure 3:**
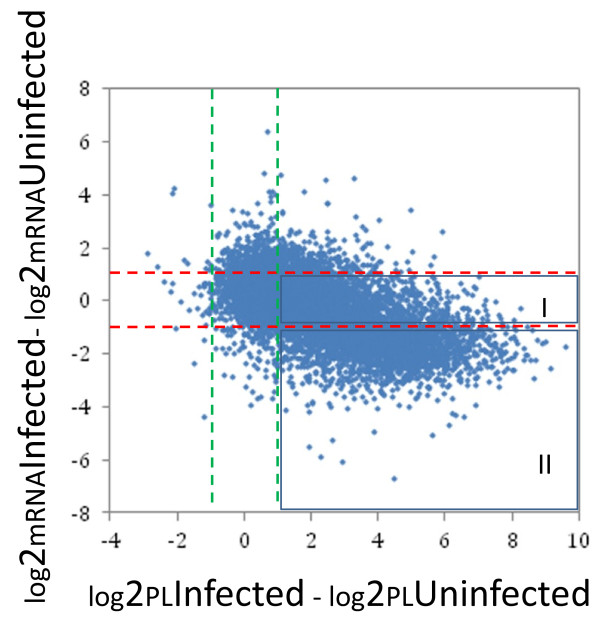
**Comparison of mRNA abundance and mRNA polysomal association between *P. falciparum* -challenged and control mosquitoes.** The difference in transcript levels (log_2_mRNA_Infected_- log_2_mRNA_Uninfected_) was plotted with the difference in translation state (log_2_PL_Infected_ - log_2_PL_Uninfected_) at around 24 h post blood meal in mosquitoes that were fed on *P. falciparum*-infected and -uninfected blood. Cut-off values (−1.0 and 1.0) for the change in transcript levels and translation state is depicted as red and green dashed lines, respectively.

It is conceivable that some of changes in PL values simply reflect up- or down-regulation at the transcriptional level. The central concept of translational regulation is that cellular gene expression is governed by the efficiency of translation of a given mRNA in the absence of a corresponding change in steady-state mRNA levels [16]. In order to uncover mosquito genes that are regulated by translational mechanisms after exposure to *Plasmodium* parasites, we plotted the difference in mRNA association with polysomes (log_2_PL_Infected_ - log_2_PL_Uninfected_) between the *Plasmodium*-challenged and control mosquitoes against the difference in cellular transcript levels (log_2_mRNA_Infected_- log_2_mRNA_Uninfected_) for every detectable mRNA. As shown in Figure 3, the dots in rectangle I represent transcripts that show no significant difference in total cellular abundance between the two mosquito groups, but vary considerably in polysomal association, indicating that expression of those genes is up-regulated at the translational level. The transcript levels of some other mRNA species (dots in rectangle II) decrease in the *Plasmodium*-challenged mosquitoes, while the mRNA polysomal recruitment even increases in these mosquitoes, again demonstrating the translational enhancement in the infected mosquitoes.

**Figure 4:**
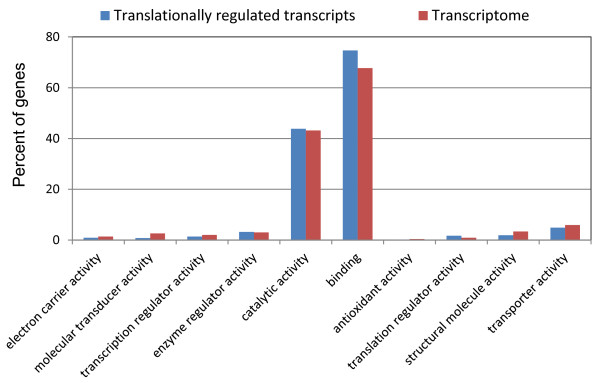
**Annotation of translationally regulated genes.** The identified translationally regulated genes are grouped by molecular function based on the *Anopheles gambiae* GO annotation information (www.vectorbase.org). GO identities were imported into WEGO (http://wego.genomics.org.cn/cgi-bin/wego/index.pl; [17]) to provide a list of categorized functions. Percentages of genes belonging to each category are shown in y-axis. For comparison, all the genes detected in the unfractionated RNA samples (transcriptome) are also included in this analysis.

Among the 1170 transcripts showing significantly increased association with polysomes, the steady-state mRNA levels of 160 transcripts also increased after *Plasmodium* infection (Table 2). The majority of the transcripts (905) remained at relatively constant level while the amount of mRNA for 105 genes went down in the *Plasmodium*-challenged mosquitoes. For the 7 transcripts exhibiting decreased association with polysomes, their steady-state mRNA levels either showed no significant change in total cellular abundance or moved in opposite direction. Together, we have identified 1017 transcripts in the midgut of *An. gambiae* that are primarily regulated at translational level after ingestion of *P. falciparum*-infected blood. Proteins encoded by these transcripts are predicted to have diverse molecular functions (Figure 4). Compared with the transcriptome in the midgut, overrepresented or underrepresented gene ontologies were not found in the translationally regulated genes.

**Table 2:** **Numbers of *An. gambiae* transcripts showing enhanced polysomal association after feeding on *P. falciparum* -infected blood**

	**Steady-state mRNA levels**		
	**Significantly increased**	**Unchanged**	**Significantly decreased**
Transcripts showing significantly increased polysome loading (1170 in total)	160	905	105
Transcripts showing significantly decreased polysome loading (7 in total)	2	5	0

The list includes transcripts showing enhanced polysomal association [(log_2_PL_Infected_ - log_2_PL_Uninfected_) ≥1, *p* < 0.05] or decreased association [(log_2_PL_Infected_ - log_2_PL_Uninfected_) ≤ −1, *p* < 0.05] in response to *Plasmodium* infection. Unchanged steady state mRNA levels are defined as within the cut-off [1 ≥ (log_2_mRNA_Infected_- log_2_mRNA_Uninfected_) ≥ −1].

### Translational regulation of immune-related genes in mosquito defense reactions against invading *P. falciparum*

In *An. gambiae*, twenty-five immune-related genes displayed a significantly higher polysomal association and therefore more active translation during infection (Table 3). These genes are implicated in different pathways of mosquito innate immunity [18,19]. Five genes (APG4B, APG6, APG7A, APG8 and APG18B) are involved in autophagy. Seven genes (CLIPA6, CLIPA7, CLIPA14, CLIPB17, CLIPC3, SRPN1 and SPRN10) encode Clip-domain serine proteases and serine protease inhibitors that are presumably involved in activation cascades. Two genes (HPX8 and CuSOD2) encode enzymes that catalyze generation and detoxification of reactive oxygen species, respectively. Three genes (CACTUS, CASPAR and REL2) are components of Toll and IMD immune signalling pathways. Five genes (DCR1, DCR2, DROSHA, RM62F and SPNE) encode key proteins in the small RNA regulatory pathway.

**Table 3:** **Translationally regulated immune-related genes in response to *P. falciparum* infection**

**Ensembl Gene ID**	**ImmunoDB Identity**	**Polysome loading**		**Transcript abundance**	
		**Fold change (log**_**2**_**)**	**P-value**	**Fold change (log**_**2**_**)**	**P-value**
AGAP005910	APG18B	2.080	0.020	−0.812	0.135
AGAP008497	APG4B	3.183	0.002	−0.881	0.199
AGAP003858	APG6	2.031	0.014	−0.269	0.614
AGAP008637	APG7A	2.953	0.000	0.169	0.853
AGAP002685	APG8	1.606	0.048	−0.425	0.249
AGAP007938	CACTUS	2.229	0.041	0.041	0.953
AGAP006473	CASPAR	4.273	0.021	−1.055	0.288
AGAP000830	CASPS7	2.563	0.012	0.764	0.487
AGAP011788	CLIPA14	4.384	0.017	−0.078	0.926
AGAP011789	CLIPA6	2.312	0.009	0.619	0.489
AGAP011792	CLIPA7	2.336	0.025	−0.392	0.717
AGAP001648	CLIPB17	3.619	0.020	0.494	0.235
AGAP004318	CLIPC3	3.574	0.024	0.842	0.331
AGAP005234	CuSOD2	2.037	0.004	−0.531	0.141
AGAP002836	DCR1	3.632	0.000	−0.462	0.465
AGAP012289	DCR2	4.277	0.001	−1.882	0.020
AGAP008087	DROSHA	5.749	0.035	0.335	0.726
AGAP004038	HPX8	3.174	0.012	0.987	0.252
AGAP007294	IAP1	3.235	0.015	−1.360	0.029
AGAP005552	PGRPLD	1.942	0.006	−1.146	0.234
AGAP006747	REL2	1.767	0.019	−0.637	0.099
AGAP012523	RM62F	2.005	0.019	−0.446	0.478
AGAP002829	SPNE	3.042	0.047	−0.562	0.504
AGAP006909	SRPN1	2.017	0.033	0.630	0.475
AGAP005246	SRPN10	1.743	0.041	−0.869	0.119

Among 1017 transcripts that are translationally regulated after ingestion of *P. falciparum*-infected blood, 35 transcripts have putative or established immune-related function (ImmunoDB identities). In several cases, alternate splice variants of the same gene were detected. Most of the transcripts in this list displayed significant translational change when insignificant change was observed in mRNA levels [1 ≥ (log_2_mRNA_Infected_- log_2_mRNA_Uninfected_) ≥ −1]. Some transcripts (i.e. DCR2) showed significant downregulation in transcription and upregulation in translation.

**Figure 5:**
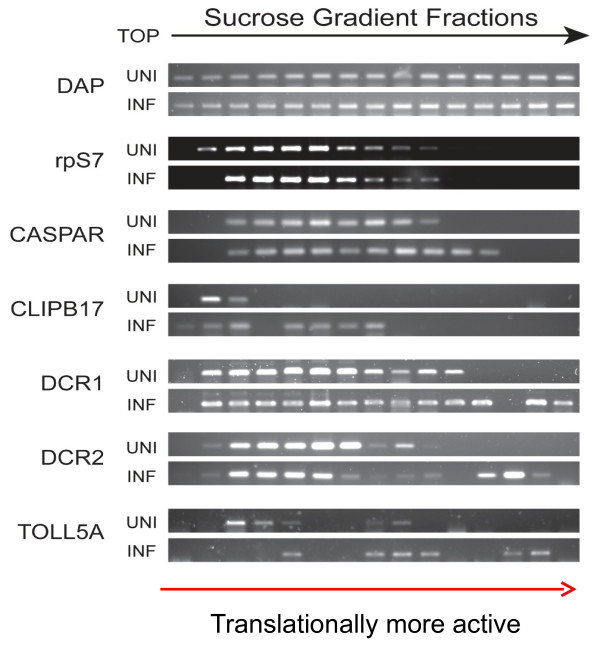
**Confirmation of polysome profiling with RT-PCR.** In an independent *P. falciparum* infection experiment, RNA samples collected from individual sucrose gradient fractions were not pooled together. RT-PCR was performed to display the distribution of mRNA in each fraction. Due to the presence of heparin in the extraction buffer, all RNA samples were treated with heparinase I (Sigma H2519) for 4 hours prior to RT-PCR in a protocol based on Izraeli S, et al. [20]. INF: *P. falciparum*-challenged mosquitoes. UNI: control mosquitoes. DAP: spike-in RNA control.

We selected four genes from the list to perform detailed polysomal profiling analysis (Figure 5). The CLIPB17 transcript was barely associated with ribosomes in the uninfected mosquitoes, and mRNA polysome association was evident in the *P. falciparum*-infected mosquitoes. For CASPAR, DCR1 and DCR2, their transcripts were loaded on polysomes even in the uninfected mosquitoes. It appeared that each mRNA molecule was associated with markedly more ribosomes in the infected mosquitoes as the transcripts were shifting more toward the bottom of the sucrose gradient, suggesting that more proteins were synthesized from each mRNA template. In contrast, the housekeeping gene *rps7* did not alter its mRNA distribution within the polysome profiles in the same experiment. The result demonstrated that the identified genes were regulated at the translational level in response to *Plasmodium* infection.

## Discussion

In this study, we discovered that a plethora of mosquito genes showed no significant difference in total cellular abundance between the *Plasmodium*-infected and uninfected mosquitoes, but varied considerably in polysomal association. Translational regulation allows cells to respond swiftly to all types of environmental stimuli and to fine-tune protein levels in both time and space. For most of the genes, it is unknown whether the enhanced translation is directly related to defense responses against invading *P. falciparum*.

Some of the genes, however, have been shown previously to play pivotal roles in anti-*Plasmodium* immunity. When the midgut of *An. gambiae* was invaded by the rodent malaria parasite *Plasmodium berghei*, SRPN10 was transcriptionally activated and overexpressed SRPN10 proteins were detected in the parasite-invaded midgut epithelial cells [21]. However, the mRNA levels were not significantly changed when *An. gambiae* was infected by the human malaria parasite *P. falciparum*[21,22]. Our experiment suggests that more SRPN10 proteins are still synthesized in the midgut of the infected mosquitoes as a result of enhanced translation.

Rel1 and Rel2, two NF-κB-like transcription factors, are key regulators of the mosquito immune response. Rel1 plays a critical role in controlling resistance of *An. gambiae* to the rodent malaria parasite *P. berghei*, while Rel2 seems to provide more protection against the human malaria parasite *P. falciparum*[23,24]*.* Rel1 and Rel2 are controlled by the negative regulators Cactus and Caspar, respectively. Our analysis suggested that Rel2, Cactus and Caspar were all translationally upregulated in the *P. falciparum*-infected mosquitoes. In future studies it will be interesting to find out whether over-expression of these proteins is restricted in the parasite-invaded midgut epithelial cells or occurs in all the epithelial cells. This may help to explain why expression of Rel2 and its inhibitor are both enhanced in response to *Plasmodium* infection.

We found significant up-regulation at the translational level in multiple proteins (Dcr1, Dcr2, and Drosha) that are required for biogenesis of miRNA and siRNA. The siRNA pathway is essential to the survival of mosquitoes infected with Sindbis virus and West Nile virus [25,26]. The miRNA pathway has previously been implicated in *An. gambiae* when infected with *Plasmodium*[27] and in Culicine mosquitoes infected with viral invaders [28]. Winter et al. found that *P. berghei* infection was significantly enhanced by knockdown of key components of the miRNA pathway (Dcr1, Ago1, and Drosha), suggesting a role for these proteins in the response to infection [27]. This is the first time, to our knowledge, that mosquito miRNA processing proteins have been found to be regulated at the translational level in response to *P. falciparum* infection in *An. gambiae*.

Additional proteins showing significant translational change include Spindle E (spnE), which is involved in RNA interference [29]. The predominantly upregulated translation that we have observed in this experiment and the presumably enhanced RNA interference activity seem to crease a paradox. In a preliminary study, we have observed significant increase of several mosquito miRNAs in the *P. berghei*-infected mosquitoes as compared with the uninfected control. Studies are ongoing to determine functions of those miRNAs and to identify their mRNA targets in the mosquito midgut.

Translation of mRNA transcripts that we identified in this study is modulated without affecting the translational status of the cellular transcriptome as a whole. Several regulatory mechanisms could lead to the enhanced polysome association of mRNA: subcellular translocation may render some mRNA more accessible to ribosomes; particular RNA sequence motifs that are present in the 5′ and/or 3′ untranslated regions (UTRs) of the target mRNA are recognized by sequence-specific RNA-binding proteins. Are all the identified transcripts regulated by the same mechanism? A follow-up question is, what signal initiates the translational regulation? The *CTRP* knockout mutant strain of *P. falciparum* has impaired locomotion and is unable to invade the midgut epithelium [30]. Comparing mRNA translational states between mosquitoes that are infected by ookinetes of the wild-type *Plasmodium* or *CTRP* knockout *Plasmodium* mutant will reveal whether the translational regulation is triggered by ookinete invasion of the mosquito midgut.

This study takes a snapshot at 22–26 h after blood feeding, and demonstrates that both transcriptional and translational regulation of mosquito genes takes place when *Plasmodium* parasites invade the mosquito midgut. In order to provide a comprehensive view of the mode of gene regulation, more experiments need to be conducted with midguts and some other tissues (i.e. the fat bodies) at various time points after an infected blood meal. Furthermore, it is imperative to examine translational regulation in natural vector-parasite combinations since co-evolution and natural selection has made the mosquito-*Plasmodium* interactions vary among different vector-parasite combinations, or even between different geographical strains of the same species. Comparing gene expression in the *Plasmodium*-refractory and susceptible mosquitoes taken recently from an epidemic region in Africa may lead to identification of mosquito factors that determine the ability of mosquitoes to transmit the parasites.

## Conclusions

We report a previously neglected aspect of gene expression regulation in the midgut of *An. gambiae* mosquitoes after ingestion of a *Plasmodium*-infected blood meal. Compared with the mosquitoes fed on uninfected blood, a large group of genes in the infected mosquitoes exhibit a redistribution of their mRNA transcripts between monosomes (or ribonucleoprotein particles) and translationally active polysomes, without a concomitant change in the steady-state mRNA levels. This finding supports our hypothesis that the anti-malarial response of *Anopheles gambiae* occurs at both the transcriptional and translational level. Elucidating the profound impact on translation of mosquito mRNA will extend knowledge of the molecular interaction between malaria parasites and their mosquito hosts. Genes for which mRNA translation is affected by *Plasmodium* infection may have been previously deemed “irrelevant” simply because their transcription is not altered by exposure to the parasites. Functions of genes identified in this project will be further explored in future studies to reveal new targets that can be employed to reduce mosquito vector competence.

## Methods

### Gametocyte production and bloodfeeding

*P. falciparum* gametocyte cultures for infecting *Anopheline* mosquitoes were generated largely based upon the protocol of Looker and Taylor-Robinson [31]. Gametocyte cultures were initiated from asexual *P. falciparum* cultures (3D7G) following daily dilution with fresh human red blood cells and medium, with a starting parasitemia typically of 3-5% and a hematocrit of 5%. Gametocytemia was monitored during the course of each culture. At 17 days after seeding, an optimal mixture of male and female stage V gametocytes were present. Gametocytes were harvested and used immediately in bloodfeeding of mosquitoes.

Female adult *An. gambiae* (G3 strain) mosquitoes were maintained on 10% sucrose at 27 °C/80% humidity. Prior to bloodfeeding, the mosquitoes (4–6 days post-eclosion) were maintained overnight on water only. Bloodfeeding was carried out using a Hemotek 5 W1 system and collagen Hemotek feeding membranes (Discovery Workshops, Lancashire, England). Mosquitoes were allowed to feed for 15 minutes. This resulted in bloodfeeding of approximately 80-90% of available *Anopheles gambiae* females. A control group of mosquitoes were fed on uninfected blood and serum by essentially the same method.

### Polysomal RNA preparation

Feeding on infected and uninfected blood was scheduled 5 hours apart. Two hundred female mosquitoes were dissected for each group at 22–26 hours post blood meal (PBM) to obtain midguts. Dissections started at 22 h PBM and ended at 26 h PBM for both groups at each experiment. The samples were flash frozen in liquid nitrogen and stored at −80 °C until processed. Frozen samples were ground using a mortar and pestle, homogenized in 1 ml lysis buffer (15 mM Tris–HCl, pH 8.0, 300 mM NaCl, 5 mM MgCl_2_, 0.5 mM DTT, 0.1 mg/ml cycloheximide, 1 mg/ml heparin, 1% triton X-100, 0.2 U/ml RNase inhibitor) [32]. The extracts were spun 15 minutes at 14,000 rpm at 4 °C to remove cell debris and nuclei. An aliquot of the supernatant was saved for analysis of total cellular mRNA. The rest of the supernatant was directly applied to the top of a 10-60% linear sucrose gradient that was prepared as described by Arava [33], containing 20 mM Tris–HCl (pH 8), 140 mM KCl, 5 mM MgCl_2_, 0.5 mM DTT, 0.1 mg/ml cycloheximide, and 0.5 mg/ml heparin. Centrifugation was carried out using an SW41-Ti rotor at 116,000 × g for 3 hours at 4 °C. Following spinning, gradient fractions were collected immediately using a Density Gradient Fractionation System (Brandel, Gaithersburg, MD) with simultaneous recording of absorbance profiles at 254 nm.

Following fractionation, non-polysomal aliquots (as shown in Figure 1) were pooled together into one sample, and polysomal aliquots were pooled into a second sample. The fraction between was discarded to ensure good separation. RNA samples from three independent infection experiments were collected. For each set, we had six RNA samples: polysomal and nonpolysomal RNAs of the infected mosquitoes, polysomal and nonpolysomal RNAs of the uninfected controls, and total cellular mRNAs of the infected and uninfected mosquitoes. A fourth set of samples were maintained as individual fractions for qRT-PCR analysis of specific transcripts.

### Oocyst observation

At 7–9 days after blood feeding, 10–30 females from the same cohorts that had been used earlier for RNA collection were dissected for midguts to estimate the efficiency of the *P. falciparum* infection. Midguts were placed into a well of a 24-well plate with 1 ml of 1% mercurochrome for 10 minutes, followed by multiple rinses with sterile water and 5 minutes incubation to remove residual mercurochrome. *P. falciparum* oocysts were visualized under a Zeiss fluorescent microscope. The results are shown in Additional file 3: Figure S1.

### RNA Isolation and sequencing preparation

RNA was extracted from the fractionated and unfractionated samples using Trizol reagent (Invitrogen, Carlsbad, CA). Sample preparation for Illumina-based mRNA sequencing was conducted based upon the manufacturer’s protocols “mRNA Sequencing Sample Preparation Guide” and “Preparing Samples for Multiplexed Paired-End Sequencing” (Part# 1005361Rev B, and Part# 1004898Rev D, Illumina, Inc., San Diego, CA). For each set, six cDNA libraries derived from the six RNA samples were uniquely tagged with index sequences at the PCR stage of sample preparation. Six libraries were pooled together and sequenced in a single lane of a flow cell. 76-cycle single read Illumina sequencing was conducted at the Virginia Bioinformatics Institute (VBI, Blacksburg, VA).

### Data analysis of the deep sequencing results

Sequencing reads were sorted into separate files according to their barcode, and linker sequences were removed. Each file was then converted into fasta format, and blasted against the annotated *An. gambiae* transcripts (CDNA-ALL.AgamP3.4.fa; from Vectorbase) with an e-value cutoff set to −20. The number of hits for each transcript was compiled in a list with a master table generated that showed the numbers of reads for each transcript in each sample. All RNA-seq data is deposited in Gene Expression Omnibus [GEO: GSE38707].

In order to obtain an accurate picture of the change in mRNA abundance, we eliminated transcripts from further consideration when the total hits in the paired polysomal and nonpolysomal pools of either infected mosquitoes or uninfected control were below 20 reads. We reasoned that levels of these transcripts were barely above the threshold of sequencing detection, and the numbers of reads might not accurately reflect their true abundance.

RNA-seq data for the unfractionated RNA samples (total cellular RNA) were normalized with *rps-7*, a commonly used internal housekeeping control gene encoding 40 S ribosomal protein S7 [34,35]. As shown in Figure 5, the distribution of *rps-7* mRNA in sucrose gradients was quite similar between the *P. falciparum*-infected and uninfected mosquitoes. Polysomal and non-polysomal RNA samples were each separately normalized against *rps-7* in the corresponding sucrose gradient fractions.

The portion of each transcript associated with polysomes (polysome loading, PL) in each sample was assessed from the number of reads for polysomal (P) and non-polysomal (NP) as follows: PL = P/(NP + P). Averages across the three replicate samples for each transcript were obtained, and log_2_ transformations of these averages were performed. To evaluate the change in translational state for every transcript, the log_2_ PL was compared between the *P. falciparum*-infected mosquitoes and the uninfected control. Unpaired two-tailed *t*-tests were conducted as described by Kawaguchi et al. [9]. Transcripts with *p*-values below 0.05 were examined further.

### Quantitative real-time RT-PCR (qRT-PCR)

To validate the polysomal association data, we carried out qRT-PCR analysis with aliquots of the pooled polysomal and non-polysomal samples that were used in RNA-seq analysis. Sixteen randomly selected transcripts were measured using the Invitrogen SYBR GreenER kit (Invitrogen, Carlsbad, California) according to manufacturer’s protocols, with an ABI Prism 7300 Real Time PCR System (Applied Biosystems, Carlsbad, California). The sequences of the primers used are listed in Additional file 2: Table S2.

## Competing interests

The authors declare that they have no competing interests.

## Authors' contributions

EAM, ML and JZ carried out experimental procedures. EAM generated *Plasmodium falciparum* gametocytes from *in vitro* culture. EAM, ZT and JZ analyzed the RNA-seq data. EAM and JZ wrote the article. All authors read and approved the final manuscript.

## Supplementary Material

Additional file 1 **Table S1.**Analysis of the steady-state mRNA levels and mRNA association with polysomes during *Plasmodium* infection. (XLSX 470 kb) Click here for file

Additional file 3 **Figure S1.***Plasmodium falciparum oocyst counts in Anopheles gambiae at 7–9 days post blood feeding.* Midguts were examined in 56 female mosquitoes that had been fed on *P. falciparum*-infected blood. Each “*” represents one mosquito. 64.3% of the mosquitoes were infected. It is possible that the actual infection rate was higher as we possibly did not remove away some of the unfed mosquitoes. The infection rate represents a conservative estimate. The average infected midgut with oocysts had 2.22 oocysts. The image on the right panel shows oocysts that we observed in a representative mosquito. (JPEG 985 kb) Click here for file

Additional file 2 **Table S2.**Primers used in qRT-PCR. Click here for file

## References

[B1] Murray CJ Rosenfeld LC Lim SS Andrews KG Foreman KJ Haring D Fullman N Naghavi M Lozano R Lopez AD Global malaria mortality between 1980 and 2010: a systematic analysis Lancet 2012 379 9814 413 431 10.1016/S0140-6736(12)60034-8 22305225

[B2] Sinden RE Alavi Y Raine JD Mosquito–malaria interactions: a reappraisal of the concepts of susceptibility and refractoriness Insect Biochem Mol Biol 2004 34 7 625 629 10.1016/j.ibmb.2004.03.015 15242703

[B3] Whitten MM Shiao SH Levashina EA Mosquito midguts and malaria: cell biology, compartmentalization and immunology Parasite Immunol 2006 28 4 121 130 10.1111/j.1365-3024.2006.00804.x 16542314

[B4] Cirimotich CM Dong Y Garver LS Sim S Dimopoulos G Mosquito immune defenses against Plasmodium infection Dev Comp Immunol 2010 34 4 387 395 10.1016/j.dci.2009.12.005 20026176 PMC3462653

[B5] Blandin SA Marois E Levashina EA Antimalarial responses in Anopheles gambiae: from a complement-like protein to a complement-like pathway Cell Host Microbe 2008 3 6 364 374 10.1016/j.chom.2008.05.007 18541213

[B6] Dong Y Aguilar R Xi Z Warr E Mongin E Dimopoulos G Anopheles gambiae immune responses to human and rodent Plasmodium parasite species PLoS Pathog 2006 2 6 e52 10.1371/journal.ppat.0020052 16789837 PMC1475661

[B7] Kumar S Christophides GK Cantera R Charles B Han YS Meister S Dimopoulos G Kafatos FC Barillas-Mury C The role of reactive oxygen species on Plasmodium melanotic encapsulation in Anopheles gambiae Proc Natl Acad Sci USA 2003 100 24 14139 14144 10.1073/pnas.2036262100 14623973 PMC283559

[B8] Vlachou D Schlegelmilch T Christophides GK Kafatos FC Functional genomic analysis of midgut epithelial responses in Anopheles during Plasmodium invasion Curr Biol 2005 15 13 1185 1195 10.1016/j.cub.2005.06.044 16005290

[B9] Kawaguchi R Girke T Bray EA Bailey-Serres J Differential mRNA translation contributes to gene regulation under non-stress and dehydration stress conditions in Arabidopsis thaliana Plant J 2004 38 5 823 839 10.1111/j.1365-313X.2004.02090.x 15144383

[B10] Koritzinsky M Magagnin MG van den Beucken T Seigneuric R Savelkouls K Dostie J Pyronnet S Kaufman RJ Weppler SA Voncken JW Gene expression during acute and prolonged hypoxia is regulated by distinct mechanisms of translational control EMBO J 2006 25 5 1114 1125 10.1038/sj.emboj.7600998 16467844 PMC1409715

[B11] Qin X Ahn S Speed TP Rubin GM Global analyses of mRNA translational control during early Drosophila embryogenesis Genome Biol 2007 8 4 R63 10.1186/gb-2007-8-4-r63 17448252 PMC1896012

[B12] Iguchi N Tobias JW Hecht NB Expression profiling reveals meiotic male germ cell mRNAs that are translationally up- and down-regulated Proc Natl Acad Sci USA 2006 103 20 7712 7717 10.1073/pnas.0510999103 16682651 PMC1472510

[B13] Provenzani A Fronza R Loreni F Pascale A Amadio M Quattrone A Global alterations in mRNA polysomal recruitment in a cell model of colorectal cancer progression to metastasis Carcinogenesis 2006 27 7 1323 1333 10.1093/carcin/bgi377 16531451

[B14] Xi Y Nakajima G Schmitz JC Chu E Ju J Multi-level gene expression profiles affected by thymidylate synthase and 5-fluorouracil in colon cancer BMC Genomics 2006 7 68 10.1186/1471-2164-7-68 16584549 PMC1448211

[B15] Melamed D Eliyahu E Arava Y Exploring translation regulation by global analysis of ribosomal association Methods 2009 48 3 301 305 10.1016/j.ymeth.2009.04.020 19426805

[B16] Holcik M Sonenberg N Translational control in stress and apoptosis Nat Rev Mol Cell Biol 2005 6 4 318 327 10.1038/nrm1618 15803138

[B17] Ye J Fang L Zheng H Zhang Y Chen J Zhang Z Wang J Li S Li R Bolund L WEGO: a web tool for plotting GO annotations Nucleic Acids Res 2006 34 Web Server issue W293 W297 16845012 10.1093/nar/gkl031 PMC1538768

[B18] Lemaitre B Hoffmann J The host defense of Drosophila melanogaster Annu Rev Immunol 2007 25 697 743 10.1146/annurev.immunol.25.022106.141615 17201680

[B19] Marois E The multifaceted mosquito anti-Plasmodium response Curr Opin Microbiol 2011 14 4 429 435 10.1016/j.mib.2011.07.016 21802348

[B20] Izraeli S Pfleiderer C Lion T Detection of gene expression by PCR amplification of RNA derived from frozen heparinized whole blood Nucleic Acids Res 1991 19 21 6051 10.1093/nar/19.21.6051 1719488 PMC329069

[B21] Danielli A Barillas-Mury C Kumar S Kafatos FC Loukeris TG Overexpression and altered nucleocytoplasmic distribution of Anopheles ovalbumin-like SRPN10 serpins in Plasmodium-infected midgut cells Cell Microbiol 2005 7 2 181 190 15659062 10.1111/j.1462-5822.2004.00445.x

[B22] Tahar R Boudin C Thiery I Bourgouin C Immune response of Anopheles gambiae to the early sporogonic stages of the human malaria parasite Plasmodium falciparum EMBO J 2002 21 24 6673 6680 10.1093/emboj/cdf664 12485988 PMC139085

[B23] Garver LS Dong Y Dimopoulos G Caspar controls resistance to Plasmodium falciparum in diverse anopheline species PLoS Pathog 2009 5 3 e1000335 10.1371/journal.ppat.1000335 19282971 PMC2647737

[B24] Mitri C Jacques JC Thiery I Riehle MM Xu J Bischoff E Morlais I Nsango SE Vernick KD Bourgouin C Fine pathogen discrimination within the APL1 gene family protects Anopheles gambiae against human and rodent malaria species PLoS Pathog 2009 5 9 e1000576 10.1371/journal.ppat.1000576 19750215 PMC2734057

[B25] Brackney DE Beane JE Ebel GD RNAi targeting of West Nile virus in mosquito midguts promotes virus diversification PLoS Pathog 2009 5 7 e1000502 10.1371/journal.ppat.1000502 19578437 PMC2698148

[B26] Myles KM Wiley MR Morazzani EM Adelman ZN Alphavirus-derived small RNAs modulate pathogenesis in disease vector mosquitoes Proc Natl Acad Sci USA 2008 105 50 19938 19943 10.1073/pnas.0803408105 19047642 PMC2604946

[B27] Winter F Edaye S Huttenhofer A Brunel C Anopheles gambiae miRNAs as actors of defence reaction against Plasmodium invasion Nucleic Acids Res 2007 35 20 6953 6962 10.1093/nar/gkm686 17933784 PMC2175301

[B28] Skalsky RL Vanlandingham DL Scholle F Higgs S Cullen BR Identification of microRNAs expressed in two mosquito vectors, Aedes albopictus and Culex quinquefasciatus BMC Genomics 2010 11 119 10.1186/1471-2164-11-119 20167119 PMC2834634

[B29] Kennerdell JR Yamaguchi S Carthew RW RNAi is activated during Drosophila oocyte maturation in a manner dependent on aubergine and spindle-E Genes Dev 2002 16 15 1884 1889 10.1101/gad.990802 12154120 PMC186417

[B30] Templeton TJ Kaslow DC Fidock DA Developmental arrest of the human malaria parasite Plasmodium falciparum within the mosquito midgut via CTRP gene disruption Mol Microbiol 2000 36 1 1 9 10.1046/j.1365-2958.2000.01821.x 10760158

[B31] Looker M Taylor-Robinson AW Kirsten Moll IL, Hedvig P, Artur S, Mats W Cultivation of Plasmodium falciparum gametocytes for mosquito infectivity studies Methods In Malaria Research 2008 5 MR4/ATCC, Manassas, Virginia

[B32] Bian G Raikhel AS Zhu J Characterization of a juvenile hormone-regulated chymotrypsin-like serine protease gene in Aedes aegypti mosquito Insect Biochem Mol Biol 2008 38 2 190 200 10.1016/j.ibmb.2007.10.008 18207080 PMC2253661

[B33] Arava Y Isolation of polysomal RNA for microarray analysis Methods Mol Biol 2003 224 79 87 12710667 10.1385/1-59259-364-X:79

[B34] Hess AM Prasad AN Ptitsyn A Ebel GD Olson KE Barbacioru C Monighetti C Campbell CL Small RNA profiling of Dengue virus-mosquito interactions implicates the PIWI RNA pathway in anti-viral defense BMC Microbiol 2011 11 45 10.1186/1471-2180-11-45 21356105 PMC3060848

[B35] Liu C Pitts RJ Bohbot JD Jones PL Wang G Zwiebel LJ Distinct olfactory signaling mechanisms in the malaria vector mosquito Anopheles gambiae PLoS Biol 2010 8 8 10.1371/journal.pbio.1000467 PMC2930861 20824161

