# Supplementary figures and images for: Translational regulation of Anopheles gambiae mRNAs in the midgut during Plasmodium falciparum infection
Source: BMC Genomics. 2012 Aug 2;13:366. doi: 10.1186/1471-2164-13-366 (PMC3443010; doi:10.1186/1471-2164-13-366)

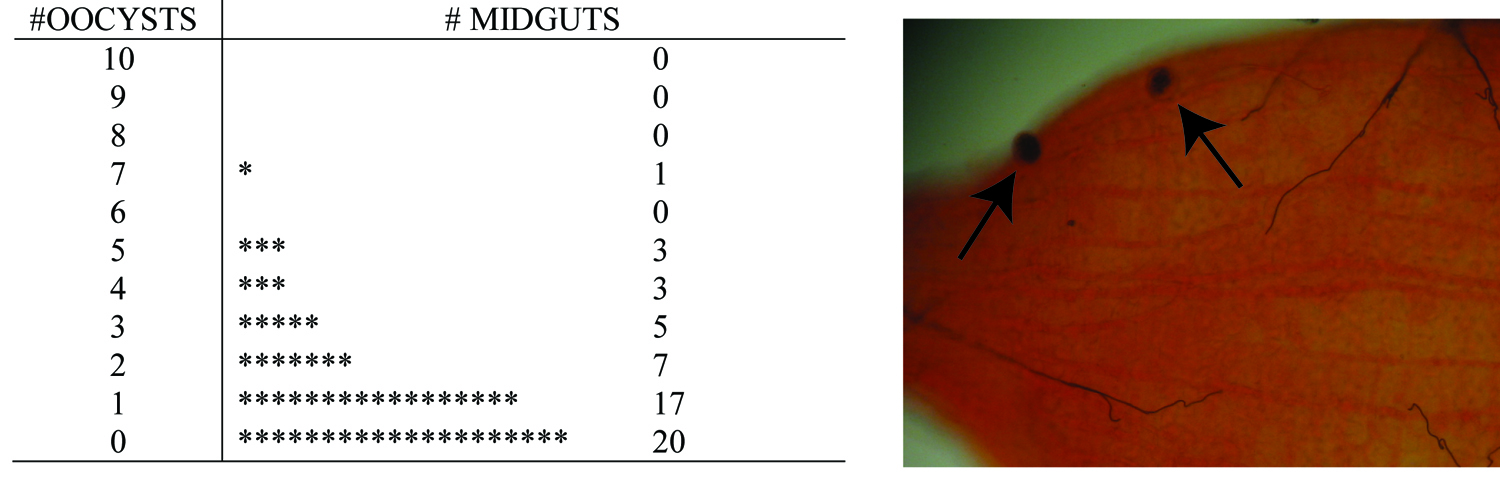

Supplement: Additional file 3 — Figure S1.Plasmodium falciparum oocyst counts in Anopheles gambiae at 7–9 days post blood feeding. Midguts were examined in 56 female mosquitoes that had been fed on P. falciparum-infected blood. Each “*” represents one mosquito. 64.3% of the mosquitoes were infected. It is possible that the actual infection rate was higher as we possibly did not remove away some of the unfed mosquitoes. The infection rate represents a conservative estimate. The average infected midgut with oocysts had 2.22 oocysts. The image on the right panel shows oocysts that we observed in a representative mosquito. (JPEG 985 kb) [file 1471-2164-13-366-S3.jpeg]
