# Supplementary material for: Translational regulation of Anopheles gambiae mRNAs in the midgut during Plasmodium falciparum infection
Source: BMC Genomics. 2012 Aug 2;13:366. doi: 10.1186/1471-2164-13-366 (PMC3443010; doi:10.1186/1471-2164-13-366)
Supplement: Additional file 2 — Table S2.Primers used in qRT-PCR. [file 1471-2164-13-366-S2.docx]

Table S2. Primers used in qRT-PCR

| Transcript Targeted | Forward Primer | Reverse Primer |
| --- | --- | --- |
| DAP | GGGATCGGAGCCATCATCGCGCCAA | TGGCGTATCTGAAGCCTGTGCAAGCG |
| rpS7 | TTCAACAACAAGAAGGCGATCA | CTTGTACACCGACGCAAAAGTG |
| Caspar | CCGCTTTTCTAAACGCTGTC | AAACAGGTTGCATGTGTGGA |
| ClipB17 | CGAAGGCATTACTTCTGCTGTACTGA | GTACGTACCGTCCTGGATTTTGACC |
| Dcr1 | CGGTGTGGGGGAGGCGGAAGATGTG | GCGTCGTCCGTCCGTCAGCTTCTCC |
| Dcr2 | GCAACGAGCTGCACCGGCACATCC | CTGGCGCACCACCTGGATCGGTGT |
